# Standardised concentrations of morphine infusions for nurse/patient-controlled analgesia use in children

**DOI:** 10.1186/s12871-019-0697-7

**Published:** 2019-02-23

**Authors:** Asia N Rashed, Cate Whittlesea, Caroline Davies, Ben Forbes, Stephen Tomlin

**Affiliations:** 1 School of Cancer & Pharmaceutical Sciences, King’s College London, 150 Stamford Street, London, SE1 9NH UK; 2 Pharmacy Department, Evelina London Children’s Hospital, Guy’s & St Thomas’ NHS Foundation Trust, Westminster Bridge Road, London, SE1 7EH UK; 3 Research Department of Practice and Policy, UCL School of Pharmacy, London, UK; 4 Paediatric Anaesthetic Department, Evelina London Children’s Hospital, Guy’s & St Thomas’ NHS Foundation Trust, London, UK

**Keywords:** Standard infusion, Morphine, Ready-to-administer infusions, Children, Pre-filled syringe, Implementation, Nurse/patient-controlled analgesia

## Abstract

**Background:**

Standardizing concentrations of intravenous infusions enables pre-preparation and is effective in improving patient safety by avoiding large deviations from the prescribed concentration that can occur when infusions are made individually in wards and theatres. The use of pre-prepared morphine standardized concentration infusions for paediatric nurse/patient-controlled analgesia (N/PCA) has not been previously investigated. We aimed to establish, implement and evaluate standardized concentrations of morphine in pre-filled syringes (PFS) for use in paediatric N/PCA.

**Methods:**

Concentrations of morphine in PFS for N/PCA were identified that accommodated dosage variation across a 1–50 kg weight range. The use of infusions in PFS was implemented and evaluated using mixed methods involved direct observation of healthcare professionals (HCPs), focus groups and failure mode and effects analysis, a HCP survey and medication incident reports analysis.

**Results:**

Standardized concentrations, 3 mg, 10 mg and 50 mg morphine in 50 mL sodium chloride 0.9%, delivered prescribed continuous and bolus doses using programmable smart pumps with variable infusion rates. During the implementation, 175 morphine pre-prepared infusions were administered to 157 children (9.4 ± 5.1 years) in theatres and wards. Time taken to set up a N/PCA was 3.7 ± 1.7 min, a reduction of one third compared with the previous system. The number of incidents associated with N/PCA infusions was reduced by 41.2%, and preparation errors were eliminated. HCPs reported using morphine PFS was an easier and safer system.

**Conclusion:**

A system using pre-prepared standardized concentrations of morphine for paediatric N/PCA was implemented successfully and sustainably.

**Electronic supplementary material:**

The online version of this article (10.1186/s12871-019-0697-7) contains supplementary material, which is available to authorized users.

## Background

Individually prepared morphine intravenous infusions have been associated with significant errors [1–3], leading to development of “ready-to-administer (RTA)” products [3–6]. Pre-prepared syringes containing standardized concentrations of drugs are recognized as important in improving patient safety by reducing medication errors [3–7]. A UK study reported aseptically prepared standardized dose-banded syringes, used with a pre-programmed safety pump was likely to reduce dosing errors in children [8].

Currently in European hospitals, morphine infusions are prepared for each paediatric patient based on their weight, using the “rule of six” formula [9]. This formula is described as: 6 x patient’s weight (kg) equals the amount of drug in milligrams that should be added to 100 mL of solution. When administered at 1 mL/h will give an infusion rate of 1 microgram/kg/min (or 60 microgram/kg/h). This is error prone, which may lead to significant over or under dosing, resulting in adverse events, e.g. hypoventilation [10] or inadequate analgesia [11, 12].

At the study hospital, a morphine prefilled syringe (PFS) system is only used to deliver continuous infusions to critically ill children in the paediatric intensive care unit (PICU) [13]. This resulted in two systems within PICU; standard concentrations for continuous infusions and individually prepared infusions for nurse/patient-controlled analgesia (N/PCA), which contributed to medication errors [11, 12].

Pre-implementation studies involving direct observation of morphine infusion preparation, with morphine concentration quantification [11] and focus groups to assess the prescribing, preparing and administering of morphine infusion for paediatric N/PCA [12], identified risks in the established process, including significant deviations in the prepared infusions from the prescribed dose. Participants suggested providing standard concentration of morphine in a RTA form would improve current practice as identified in other studies to improve the safety [3, 4, 7, 12]. It has been reported that establishing standardized morphine concentration infusions for paediatric N/PCA is complex, as both bolus and continuous doses from the same solution need to be delivered [14]. Considering this challenge this study aimed to implement quality assured standardized concentrations of paediatric N/PCA morphine infusions, supplied as PFS, ready for administration via pre-programmed safety pumps (ALaris syringe Pumps) [15] to deliver accurate bolus and continuous doses.

## Methods

This project was conducted in two stages. The pre-implementation which has previously been described [11, 12]. This paper presents the findings of the implementation stage.

*Design*: mixed methods approach.

### Intervention development

#### Establishing standard concentration

Using Excel all possible morphine concentrations from 1 mg to 50 mg in 50 mL for all possible weight ranges in children from a lower weight limit of 1 kg upwards, were proposed (ANR) and reviewed by a Consultant Paediatric Pharmacist (ST). The criteria were:

- Total daily volume of morphine infusion should not account for more than 15% of child’s total daily fluid allowance.
- Daily treatment delivered using no more than three syringes (ideally 1–2 syringes) per patient, to reduce risks and workload from multiple syringe changes;
- Manufacture a limited number of standard concentrations (safety and cost);
- Delivered by available infusion pumps for continuous infusion rate and bolus dose, with a minimum volume of 0.1 mL.

Proposed standard concentrations were reviewed and approved by the Lead Paediatric Acute Pain Consultant Anaesthetist (CD) and the Paediatric Clinical Nurse Specialist. Documentation, protocols, prescription labels (used on medication charts), and reprogramming of infusion pumps were approved (Paediatric Acute Pain Team, Paediatric Consultant Pharmacist, hospital Clinical Governance department).

The prefilled syringe containing morphine standard concentration infusions are being prepared by the centralised intravenous admixture service (CIVAS), which is a service run by the pharmacy department at our hospital.

#### Risk assessment

Failure mode and effects analysis (FMEA) was used to determine the risks/issues associated with changes to the process of delivering paediatric N/PCA infusions using morphine PFS containing standard concentration. Before implementation a multidisciplinary team (17 members; nurses, doctors and pharmacists) familiar with prescribing, preparing and administering morphine N/PCA undertook a FMEA.

The FMEA team met twice over two months (October–November 2013). Before the FMEA, the research team described the initial and final steps of the process of delivering morphine infusion for N/PCA, presented an overview of FMEA with a process scheme example [16].

Team members described the steps undertaken when prescribing, preparing, and administering morphine PFS for N/PCA use, identified potential process failures and determined severity, probability, and detectability scores for these failures. They also made recommendations to reduce the identified failures.

#### Education and training

Over three months (January–March 2014), in-house training on the use of standardized concentrations for paediatric N/PCA were provided (ANR, CD, ST) to all nurses and doctors in theatres and wards. Standard operating procedures (SOPs) for new documentation and pump programming were produced. Posters of the standard concentrations of morphine and SOPs were placed in all clinical areas.

#### Implementation

The implementation was conducted over eight months (March – November 2014):

- Stage I: dummy run of whole system on one day from theatre and transfer to a ward.
- Stage II: targeting one specific list of paediatric orthopaedic operations with the same designated consultant anaesthetist.
- Stage III: targeted lists extended to include spinal cases and cleft cases, with an increased number of anaesthetists and nursing staff participating.
- Stage IV: morphine PFS system was introduced across all clinical areas.

The staged implementation was designed to identify issues that arose when HCPs used the standardized morphine PFS for N/PCA. It also supported training of HCPs on the new system.

During implementation, all HCPs (doctors/nurses) prescribing/preparing morphine PFS for paediatric N/PCA, were observed (ANR). Data collected were patient demographics (age, sex, weight); morphine prescription details (PFS strength, N/PCA type); location; name of nurse or doctor programming and prescribing; and time spent prescribing, programming pump and administering a morphine PFS.

### Sample size consideration for observation activity.

In 2011, 896 children were administered morphine for N/PCA at the study hospital (internal report), with the increase in surgery numbers, about 1000 children/year would benefit directly from standardized morphine concentration infusions for N/PCA. With RTA a 100% reduction in preparation errors was assumed because of the elimination of the individualized preparation stage by HCPs. Based on the reported medication error percentage (1%) (internal medication error report), a sample of 150 patients was required to provide a 95% confidence interval for the true mean rate of 0.6 to 2.6%.

### Evaluation of the intervention

#### Focus groups

All HCPs (doctors/nurses) who prescribing and preparing/administering morphine N/PCA in paediatric theatres or wards were invited to attend focus groups. Signed informed consent was obtained from participants. Focus groups were conducted over 3–4 weeks (March 2015) following implementation and evaluation of the morphine PFS system post-implementation, to determine HCPs views, concerns and any aspects to improve the morphine PFS system.

#### Implementation across clinical areas

One week before the morphine PFS system’s hospital-wide introduction date (1st April 2015), the new system was publicized by email to staff and posters displayed in all clinical areas. All previous protocols and paperwork were removed and replaced with the new documentation.

#### Self-administered questionnaire

All HCPs (doctors/nurses*)* within paediatric theatres and wards, who prepared/administered morphine N/PCA, were surveyed 12 months after the morphine PFS system implementation to determine staff views and satisfaction with the morphine PFS system compared with the previous system (individually prepared syringes based on patient’s weight). The questionnaire was completed by HCPs over 6 weeks (April–May 2016). New staff were excluded because they had no experience of the previous system.

A structured anonymous questionnaire was developed based on previous studies [11, 12]. It included items assessing satisfaction, attitudes and views of HCPs on the recently implemented morphine PFS system together with demographic data (location and job title). This piloted questionnaire covered three themes: use; quality; and impact of the morphine PFS system on patient safety.

#### Hospital incident reports

Data of morphine N/PCA related incidents pre- and post-implementation of morphine PFS system for January 2013–December 2015 were extracted from the hospital electronic incident reporting system. Analysis of reported incidents was conducted to identify any medication related incidents and to assess the impact of implementing standard concentrations on reported error occurrence.

### Data analysis

Data was analysed using Stata 11 (StataCorp, College Station, TX, USA). Descriptive statistics were performed on data from observations, questionnaire, and medication incident reports, and presented as number, percentages and mean ± standard deviation (SD), unless otherwise specified. Chi-squared test was used for statistical significant (*p* < 0.05) for categorical variables, between wards and theatres.

Each focus group was transcribed verbatim and the anonymized transcript uploaded to QSR NVivo (V.10) software for coding and categorization to identify themes. Qualitative content analysis was used with five main themes being set a priori and supplemented by emergent subthemes identified during analysis. An iterative approach involving constant comparison was employed where all data relating to each theme were constantly revisited after initial coding. Coding frames were prepared and framework analysis created by ANR and checked by ST independently.

Questionnaire data: the 5-point Likert questions were grouped to three groups; “strongly agree” and “agree” where considered as “agreement”, likewise “strongly disagree” and “disagree” were considered “disagreement”, and “neutral” (neither agree nor disagree).

## Results

### Intervention development

#### Establishing standard concentration

Three standard concentrations were established for paediatric N/PCA use (Table 1). Protocols and prescription labels were also developed (Additional file 1: Figure S1-S4).

**Table 1:** Weight bands and morphine standardized prefilled syringe strengths established for N/PCAª

Weight band	Protocol	Morphine PFS strength
Weight ≤ 3.9 kg	NCA	3 mg in 50 mL Glucose 5%
Weight ≥ 4 kg – 19.9 kg	NCA	10 mg in 50 mL Sodium Chloride 0.9%
Weight ≥ 20 kg	NCA	50 mg in 50 mL Sodium Chloride 0.9%
Weight ≥ 25 kg	PCA	

^a^N/PCA: Nurse- or/ Patient-Controlled Analgesia

#### Risk assessment

Seventeen (89.5%, 17/19) HCPs participated in the FMEA meetings (Additional file 1: Table S1). FMEA identified potential failures which might occur when using morphine PFS system for N/PCA as well as recommendations to address these aspects (Table 2).

**Table 2:** Potential failures of the morphine PFS system and recommendations identified by FMEA

Potential Failure	Causes	Effects	S	P	D	RPN	Recommendations
Staff prepare N/PCA infusion from ampoule using the previous system (based on patient weight)	PFSs stock not updated quickly or prefilled syringe expired.	Possible of delay in patient receiving morphine dose as individualized syringe can’t be administered using the standard syringe programs on the pump, as protocols on the pump are for standard concentrations only.	2	8	1	16	− Nurse review stock levels − Protocols need to be clear about preparing standard concentrations from ampoules in ward in emergency (pharmacy)
Run out-of-stock quickly at ward level	No enough space to store PFSs and drug room temperature is above recommended temp, > 25 °C.	PFSs not available when required. Delays in patient receiving morphine injection	4	10	1	40	− Additional air conditioning in drug storage area (matrons) − Use Omnicell (electronic storage cabinets) for storage as temperature controlled (pharmacy)
Run out-of-stock in paediatric pharmacy dispensary area	No enough space in paediatric pharmacy dispensary area to accommodate large number of the three strengths.	Limited number of PFSs stored at wards level.	4	6	1	24	− Increase stock levels at paediatric Pharmacy − Consider using Omincell for CD storage in paediatric pharmacy dispensary area, wards, and theatres (Pharmacy)
Choosing the wrong strength of the PFS	Picking syringe by label, not by barcode.	Wrong dose given to patient	10	3	2	60	− Separate storage for each strength, with clear labelling, on the wards/theatre (Pharmacy) − Write weight between brackets in large font on the syringe label (pharmacy manufacture) − Introduce the use of barcodes for syringe’s label, prescriptions’ label.
Syringe Drive procedure incompatibility with manually made up solution in Emergency Department	Misinterpretation of fall-back case – Is manually mixing equivalent to PFSs, or follow previous procedure?	Would have to select standard concentrations	5	1	2	10	Protocols and SOPs on how to use standard concentrations should be made clear to all clinical areas, including Emergency Department. (Pharmacy)

FMEA: Failure Mode and Effects Analysis; N/PCA: Nurse- or/ Patient-Controlled Analgesia; PFS: prefilled syringe; SOPs: standard operating procedures; S=Severity; P=Probability; D = Detectability; RPN: risk priority number calculated as RPN = S x P x D; CD: controlled drug

The two aspects with the highest potential for failure were identified at ward level due to limited storage space and staff selection of the wrong strength. The risk assessment resulted in a staged implementation (I-IV).

#### Observation of HCP setting up N/PCA using morphine PFS

**Fig. 1:**
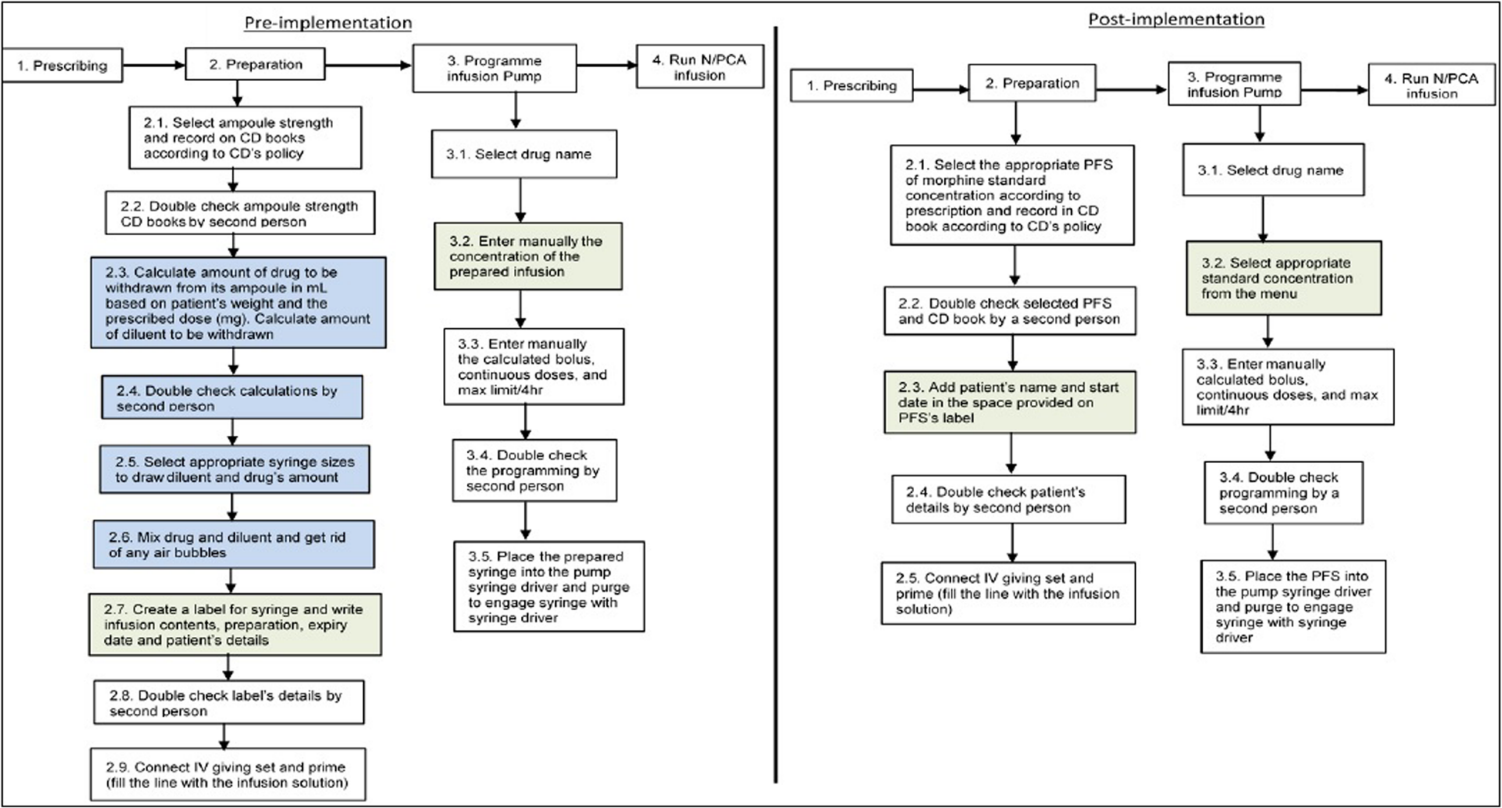
Process of prescribing, preparing and administering morphine N/PCA pre- and post-implementation of PFS containing morphine standardised concentrations at the participated hospital**.** CD: controlled drug, IV: intravenous; PFS: prefilled syringe. Highlighted steps in blue in the preparation stage were eliminated after introducing PFS system. Green boxes show the changes in those steps pre- and post-implementation

A total of 175 morphine PFS (theatres 157, wards 18) were administered to 157 children [mean (sd); age 9.4 years ±5.1, mean weight 32.4 kg ± 15.2, weight range 5–54 kg] were observed. Fig. 1 shows the pre- and post-implementation processes of setting up morphine PFS paediatric N/PCA. Using morphine PFS, resulted in fewer steps in the preparation process (5 compared to 9 steps). When programming the infusion pump, the infusion concentration was pre-set and fixed which reduced the values needed to be programmed (4 to 3 steps). Therefore, less time was required to set up each PFS.

Overall the total mean time required to set up N/PCA for a child (prescribing, programming pump/administering) was 3.7 ± 1.7 (sd) minutes. There was a significant difference between theatres and wards (theatre 3.6 ± 1.7; ward 4.7 ± 1.3 min, *p* < 0.01). This suggested that theatre staff might be faster in setting up a N/PCA for various reason; e.g. could be because of the theatre and ward’s layout, i.e. theatre have the drug, paperwork and the patient in one place. While on the ward, nurses have to be between drug room and patient bedside.

The overall time spent by HCPs using the previous system to set up N/PCA for a child has been reported [11]. There was a significant difference between the morphine PFS system and previous system (3.7 ± 1.7 vs. 11.9 ± 4.1, *P* < 0.001 and between theatres (morphine PFS system 3.6 ± 1.7; previous system 10.5 ± 3.3, *p* < 0.001) and wards (morphine PFS system 4.7 ± 1.3; previous system14.5 ± 4.0, p < 0.001).

The majority of prescriptions were for NCA use (116/175, 66.3%), of which 69% (80/116) were for strength 50 mg/50 mL and 31% (36/116) for 10 mg/50 mL. Only 59 prescriptions were for PCA using 50 mg/50 mL PFS.

### Evaluation of the intervention

#### Focus groups

Two focus groups were conducted with participants recruited from three different clinical areas; focus group 1 ward and recovery nurses (*n* = 7); focus group 2 paediatric anaesthetists (*n* = 5) from theatres.

Five main themes were identified: 1) the process of using morphine PFS system to set up N/PCA infusion; 2) impact of this system on the process of preparing/administering morphine N/PCA; 3) concerns about this system; 4) suggestions to address concerns with this system; 5) impact of this system on practice and patient care. Table 3 summarizes theme and subthemes identified.

**Table 3:** Summary of topic themes and subthemes identified from the focus groups

Theme	Subthemes
The process of setting up NCA/PCA infusion using morphine standardized concentration PFS	Prescribing - paper work
	Select PFS of the required standard concentration and check
	Programming the pump
	Double checking process
	Changing syringes on ward
Impact of the morphine PFS system on the process of preparing/administering morphine N/PCA	Faster – less time consuming
	Easier to set up No calculation of concentration Safer – less errors Eliminating any errors of how much patient is getting Less infection risks Paperwork and pump programming much easier to read
	Fixed standardized concentrations in all clinical areas Use same dose as previous system but volume vary for each patient Volume of continuous and bolus doses cannot be used as safety net as previous system
Concerns about the morphine PFS system	Risk of picking up the wrong PFS Out of stock - due to storage limit space or expiry Human error still same as previous system
Suggestions to overcome concerns and improve the morphine PFS system	Emphasize on the double checking as safety mechanism Possibility of introducing standard concentration in 50-ml vials to extend expiry and maximize stock storage Possibility of storing PFS in Omnicell to increase stock level Make up standard concentration in case of out-of-stock PFS Order before it ran-out or expired Look into using of barcoding syringe, label and prescription to avoid wrong selection of syringe
Impact of the morphine PFS system on practice and patient care	Time efficient Safer practice Less risk of errors - improve patient safety Allow focus on the patient rather than on paperwork and preparation Give more time for teaching trainee
N/PCA: Nurse- or/ Patient-Controlled Analgesia; PFS: pre-filled syringe; Omnicell: electronic storage cabinet	

All focus group participants preferred using the morphine PFS system and had positive comments. The morphine PFS system was described as easier and safer; because it eliminated errors with calculations, dose received by the patient, reduced infection risk and less time consuming. Example quotes from focus group participants;

One of the participated nurses commented that *“It* [morphine PFS system] *is much easier than starting that from the scratch … time is less than* [it] *used to be.”* While one of the anaesthetists said that morphine PFS system is *“Unquestionably quicker”.*

The risk of selecting the wrong strength of morphine PFS was the main concern raised by participants. However, it was identified that it could be mitigated by enforcing the hospital policy of double-checking IV infusions by two people. Other measures suggested were bar-coding and using electronic storage cabinets (Table 3).

### Self-administered questionnaire

A total of 125 questionnaires (62.0%, 125/200) were completed (ward = 100, theatre = 25). Most respondents (90.4%, 113/125) were satisfied using the morphine PFS system and 8.8% (11/125) were neutral. Only one respondent (0.8%), who provided no explanation, was dissatisfied (Table 4).

**Table 4:** Summary of the questionnaire results

Theme/Items	Disagree *n*(%)	Neutral *n*(%)	Agree *n*(%)
*Evaluation of the morphine PFS system for N/PCA; n (%)*			
Set up time is quicker (5–9 min)	1 (0.8)	11 (8.8)	113 (90.4)
New paperwork easier to use	2 (1.6)	26 (20.8)	97 (77.6)
If PFS out-of-stock; easier to prepare standard concentration than previous system (prepare individual syringe based on patient weight)	6 (4.8)	42 (33.6)	77 (61.6)
Little impact of distraction when setting up PFS compared to previous system	27 (21.6)	36 (28.8)	62 (49.6)
Satisfied with using morphine PFS on daily practice	1 (0.8)	11 (8.8)	113 (90.4)
*Quality of the morphine PFS system*			
Less time spend in setting up PFS is beneficial	0	5 (4.0)	120 (96)
Using PFS avoid waste of morphine ampoules	2 (1.6)	15 (12.0)	105 (84)
Prefer using aseptically prepared standard concentration to individualized preparation (mg/kg)	0	16 (12.8)	109 (87.2)
the morphine PFS system helped in making the process of administering N/PCA infusion safer	4 (3.2)	10 (8.0)	111 (88.8)
The morphine PFS system help to provide better quality of care to paediatric patients	2 (1.6)	35 (28.0)	88 (70.4)
Using PFS help in reducing incidents of injury might result from breaking ampoules	1 (0.8)	27 (21.6)	97 (77.6)
The morphine PFS system provide more accurate dosing	4 (3.2)	35 (28.0)	86 (68.8)
*Impact of the morphine PFS system on patient safety*			
Overall the morphine PFS system help to improve patient safety	0	6 (4.8)	119 (95.2)

Percentages (%) calculated out of the total number of respondents (*n* = 125)

N/PCA: Nurse- or/ Patient-Controlled Analgesia; PFS: pre-filled syringe

Overall, most respondents (95.2%, 119/125) believed that using the PFS improved patient safety and 89.6% (112/125) indicated that the morphine PFS system minimized preparation/administration errors.

Majority of respondents (90.4%, 113/125: ward = 93, theatre = 20) reported that morphine PFS system decreased drug delivery time because set up time for an N/PCA infusion was quicker compared to the previous system; with 53.1% (60/113; ward = 51, Theatre = 9) reporting it took less than 5 min to set up a PFS. Most respondents (91.2%, 114/125) suggested that pre-programming of the infusion pumps was better compared to the previous system. Only one individual (ward nurse), reported the new system was much slower (> 25 min), however no explanation was provided. Four respondents from theatre did not answer this question as they had not set up a smart pump with a prefilled syringe.

### Hospital incident reports

**Fig. 2:**
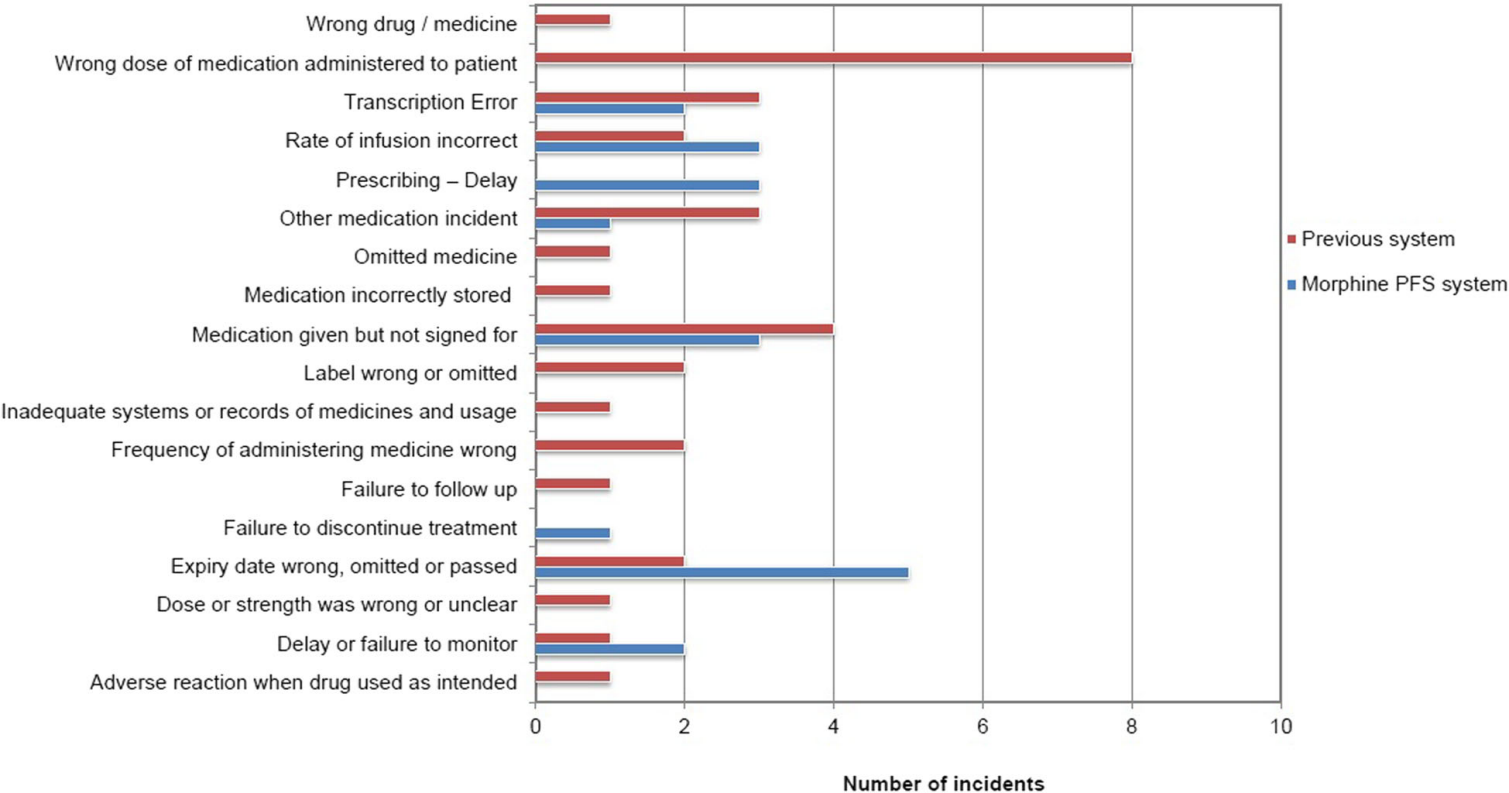
Number of morphine N/PCA related errors reported pre- and post-implementation of morphine PFS system. Low harm incidents related to previous system; “adverse reaction when drug used as intended”; “wrong dose (concentration) of drug administered”; “medicine omitted” (i.e. PCA infusion discontinued without conversion to oral dose of opioid leaving the patient in pain)

A total of 198 incident reports related to morphine reported pre-and post-implementation, were analysed, of which 54 (27.3%, 54/198) were related to N/PCA. Fig. 2 describes the reports (63%, 34/54) linked to the previous system (i.e. preparing individual syringe based on patient weight) and the reports (37%, 20/54) with the morphine PFS system.

Overall there was a 41.2% decrease in the occurrence of reported medication incidents following implementation of the standard concentrations for N/PCA use. Although this reduction was not statistically significant (*p =* 0.115), it is considered important, because incidents such as “wrong dose of medication administered to patient” and “dose or strength selected was wrong or unclear” were not reported in the morphine PFS system. They had been reported for the previous system [23.5% (8/34); 2.9% (1/34), respectively].

The incidence of “expiry date’s wrong, omitted or passed” (25%, 5/20) was higher in the morphine PFS system compared to the previous system (5.9%, 2/34).

The majority of reported incidents were reported to have “no harm” (51/54). Only three were reported as “low harm”, all related to the previous system (Fig. 2).

## Discussion

The main finding of this study is that the established standardized concentrations of morphine provided in PFS were implemented successfully across all clinical areas in our hospital to deliver N/PCA infusion; both bolus and continuous doses from the same syringe, in children. While standard concentrations of some intravenous (IV) drugs have been widely used for IV infusions in various healthcare systems [4–7], this was the first study within a paediatric hospital where standardized morphine concentrations in “ready-to-administer (RTA)” infusions were implemented to deliver paediatric N/PCA infusions.

The data derived and feedback collected provided an evidence base that encouraged HCPs’ acceptance of the change to standardized concentration infusions for paediatric N/PCA.

The new system eliminated the need to calculate the concentration and reducing the risk of microbial contamination providing a safer and more time efficient system. This was evidenced by observation, findings from focus groups and survey questionnaire. Furthermore, the pre-implementation study [11] identified that 61.5% of individually prepared syringes deviated unacceptably from the intended morphine concentration. Infusions prepared on an individual patient basis in clinical areas are prone to such errors, which are classically ‘unseen errors’ because syringe content is not routinely analysed and therefore not captured by incident reporting systems. Such errors are eliminated in the new system through batch manufactured, quality assured morphine PFS.

The main goal in implementing morphine PFS containing standard concentrations was to improve paediatric patient safety by minimizing the risk of medication errors. Twelve months following implementation, there was a reduction in medication errors compared with the pre-implementation period. Although this was not statistically significant, from clinical perspective it was considered important because the newly implemented system eliminated errors associated with infusion preparation. Medication errors associated with intravenous infusion have previously been reduced using standard concentrations [4]. Importantly, the new system did not increase other types of medication error e.g. delivery of the wrong dose. The follow-up survey demonstrated that staff perceived that the morphine PFS improved medication safety and resulted in reduced set up time.

### Limitations

The study has some limitations that need to be considered when interpreting our results. Observations of HCPs prescribing/preparing morphine PFS for N/PCA were conducted during day shifts (8 am - 5 pm) Monday to Friday. Therefore, any use of the morphine PFS system during other times was not undertaken. Whilst errors are generally under–reported [17], highlighting a process (positively or negatively) tends to lead to increased reporting [18]. Bias in reporting errors linked to the morphine PFS system might have occurred as it was well publicized. The incidents report data captured post-implementation might be relatively low due to the short period it was conducted within, therefore, it is recommended to reanalyse incidents reports over a longer period post-implementation.

The use of the lowest strength morphine PFS (3 mg/50 mL) was not observed. Finally, not all staff who participated in the implementation of the morphine PFS system attended focus groups; however, a follow-up self-administered survey, 12 months after implementation, was conducted to capture the impact of this system on HCPs practice and satisfaction.

## Conclusions

The implementation of standardized concentration of morphine infusions for N/PCA was achieved in all clinical areas in our hospital and satisfied the requirements for delivering continuous infusions and bolus doses for children weighing 1–50 kg. This led to improved patient safety in the study hospital, by reducing medication errors reported post implementation of morphine PFS system. This system supported HCPs to more safely deliver a high-risk medicine to children, resulting in a quality improvement within the healthcare system in our hospital.

## Additional files

Additional file 1: **Figure S1-S4** Morphine standard concentrations: N/PCA colour-coded protocols and medication charts’ prescription labels. **Table S1** Demographic details of the 17 HCPs who took part in the FMEA. (DOCX 3753 kb)

## Abbreviations

FMEA: Failure Mode and Effects Analysis
HCPs: Healthcare professionals
N/PCA: Nurse- or/ Patient-Controlled Analgesia
NHS: National Health Service
PFS: Pre-filled syringe
PICU: Paediatric intensive care unit
RTA: Ready-to-administer
SOPs: Standard operating procedures
