# Supplementary material for: Standardised concentrations of morphine infusions for nurse/patient-controlled analgesia use in children
Source: BMC Anesthesiol. 2019 Feb 23;19:26. doi: 10.1186/s12871-019-0697-7 (PMC6387512; doi:10.1186/s12871-019-0697-7)
Supplement: Supplementary file 1 — Figure S1-S4 Morphine standard concentrations: N/PCA colour-coded protocols and medication charts’ prescription labels. Table S1 Demographic details of the 17 HCPs who took part in the FMEA. (DOCX 3753 kb) [file 12871_2019_697_MOESM1_ESM.docx]

**Figure S1 - S4: Morphine standard concentrations: N/PCA colour-coded protocols and medication charts’ prescription labels**

Figure S1 NCA for ≤3.9 kg protocol and prescription label

Figure S2 NCA for 4- ≤19.9 kg protocol and prescription label

Figure S3 NCA for ≥20 kg protocol and prescription label

Figure S4 PCA for ≥25 kg protocol and prescription label

**Table S1 Demographic details of the 17 HCPs who took part in the FMEA**

| **Profession** | **Speciality** |
| --- | --- |
| Pharmacist | Highly specialist paediatric pharmacist |
| Pharmacist | Principal Paediatric Pharmacist |
| Pharmacist | Senior Pharmacist - Safety Injectable medicine |
| Nurse | Staff nurse – paediatric renal ward |
| Nurse | Clinical Nurse Specialist |
| Nurse | Pain nurse practitioner |
| Nurse | Clinical Governance |
| Nurse | Senior staff nurse-paediatric general medical ward |
| Nurse | Senior staff nurse – paediatric orthopaedic ward |
| Nurse | Staff nurse-Paediatric Intensive Care Unite |
| Doctor | Consultant paediatric anaesthetists – Pain lead anaesthetist |
| Doctor | Consultant paediatric anaesthetists |
| Clinical engineering | Specialist medical device management officer |
| Medical physics manager | Medical device |
| Quality assurance manager | Pharmaceutical Quality Control |
| Pharmacy Aseptic Service Manager | Aseptic Service |
| Associate Chief Pharmacist | Technical Services |

*HCPs: Healthcare Professionals
